# Whole genome sequencing enables new genetic diagnosis for inherited retinal diseases by identifying pathogenic variants

**DOI:** 10.1038/s41525-024-00391-2

**Published:** 2024-01-20

**Authors:** Xubing Liu, Fangyuan Hu, Daowei Zhang, Zhe Li, Jianquan He, Shenghai Zhang, Zhenguo Wang, Yingke Zhao, Jiawen Wu, Chen Liu, Chenchen Li, Xin Li, Jihong Wu

**Affiliations:** 1 CAS Key Laboratory of Computational Biology, Shanghai Institute of Nutrition and Health, University of Chinese Academy of Sciences, Chinese Academy of Sciences, Shanghai, China; 2 Eye Institute and Department of Ophthalmology, Eye & ENT Hospital, Fudan University, Shanghai, China; 3 NHC Key Laboratory of Myopia (Fudan University); Key Laboratory of Myopia, Chinese Academy of Medical Sciences, Shanghai, China; 4 Shanghai Key Laboratory of Visual Impairment and Restoration, Shanghai, China; 5 Computer Center, Eye & ENT Hospital, Fudan University, Shanghai, China

**Keywords:** Genetic testing, Genetic counselling

## Abstract

Inherited retinal diseases (IRDs) are a group of common primary retinal degenerative disorders. Conventional genetic testing strategies, such as panel-based sequencing and whole exome sequencing (WES), can only elucidate the genetic etiology in approximately 60% of IRD patients. Studies have suggested that unsolved IRD cases could be attributed to previously undetected structural variants (SVs) and intronic variants in IRD-related genes. The aim of our study was to obtain a definitive genetic diagnosis by employing whole genome sequencing (WGS) in IRD cases where the causative genes were inconclusive following an initial screening by panel sequencing. A total of 271 unresolved IRD patients and their available family members (*n* = 646) were screened using WGS to identify pathogenic SVs and intronic variants in 792 known ocular disease genes. Overall, 13% (34/271) of IRD patients received a confirmed genetic diagnosis, among which 7% were exclusively attributed to SVs, 4% to a combination of single nucleotide variants (SNVs) and SVs while another 2% were linked to intronic variants. 22 SVs, 3 deep-intronic variants, and 2 non-canonical splice-site variants across 14 IRD genes were identified in the entire cohort. Notably, all of these detected SVs and intronic variants were novel pathogenic variants. Among those, 74% (20/27) of variants were found in genes causally linked to Retinitis Pigmentosa (RP), with the gene *EYS* being the most frequently affected by SVs. The identification of SVs and intronic variants through WGS enhances the genetic diagnostic yield of IRDs and broadens the mutational spectrum of known IRD-associated genes.

## Introduction

Inherited retinal diseases (IRDs) are a group of severe retinal degenerative diseases. These diseases are a major cause of vision loss in children and young adults, affecting millions of people worldwide^1,2^. IRDs exhibit diversity in terms of etiology, clinical manifestations, and genetic underpinnings, with inheritance patterns that can be autosomal dominant (AD), autosomal recessive (AR), X-linked (XL), and mitochondrial inheritance^3,4^.

IRDs manifest in two primary phenotypic categories: nonsyndromic and syndromic. Nonsyndromic IRDs, making up the majority of cases, exclusively impact the eye, giving rise to conditions such as retinitis pigmentosa (RP), macular dystrophy, cone-rod dystrophy (CRD), Stargardt disease (STGD), Leber congenital amaurosis (LCA), retinoschisis, and choroideremia. Syndromic IRDs, driven by single gene mutations, affect multiple organ systems beyond the eyes and include conditions like Usher syndrome, Bardet-Biedl syndrome, Joubert syndrome, and others^3,5^. RP is the most common type of IRD, with a worldwide prevalence of about 1 in 4000^6,7^. To date, more than 300 genes have been associated with IRDs (RetNet, https://sph.uth.edu/retnet/), each harboring a spectrum of variants ranging from point mutations to extensive deletions and duplications^8^. Notably, different sets of genes have been identified as hotspot genes for single nucleotide variants (SNVs) and copy number variants (CNVs), with five genes—*ABCA4*, *USH2A*, *EYS*, *RPGR*, and *CRB1*—accounting for up to 50% of all IRD-associated SNVs, while the top four hotspot genes for pathogenic CNVs are *USH2A, EYS, PRPF31*, and *MERTK*^9,10^.

Despite substantial progress in identifying genes associated with IRDs through extensive sequencing efforts, a subset of IRD patients remain without a genetic diagnosis^11,12^. While some undiagnosed cases may stem from yet undiscovered genes, emerging evidence suggests that a significant portion of these unresolved cases are attributable to structural variants (SVs) and intronic variants affecting splicing in known IRD-causative genes, which are challenging to accurately detect using panel-based sequencing or whole exome sequencing (WES) techniques commonly employed in clinical practice^10,13,14^. Studies have shown that SVs can contribute 5% to 15% of IRD pathogenicity, while intronic variants causing aberrant mRNA splicing have also been widely confirmed as pathogenic contributors^15,16^. The identification of intronic variants within IRD genes holds particular significance for achieving precise diagnoses in IRD patients^17^. The identification of SVs and intronic variants require sequencing of noncoding regions of the genome. The panel-based sequencing and WES are limited in their ability to detect such variants, particularly those with breakpoints in intronic or intergenic regions, whereas WGS can overcome this limitation by covering all exonic and intronic regions of the genome^4,18,19^. Thus, for IRD patients who remain undiagnosed using conventional methods, WGS represents a promising avenue to enhance diagnostic accuracy.

In this study, we investigated the impact of SVs and intronic variants in known IRD genes using WGS technology in a cohort of Chinese patients with IRDs, who had previously tested negative in initial panel-based sequencing. Our analysis identified 22 SVs, 3 deep-intronic variants, and 2 non-canonical splice-site variants in 14 IRD genes, ultimately leading to a confirmed genetic diagnosis in 13% of IRD patients. Taken together, our findings underscore the crucial role of SV and intronic variants in augmenting the diagnostic yield for IRDs while broadening the mutational spectrum of IRD genes.

## Results

### Diagnostic yield

**Fig. 1:**
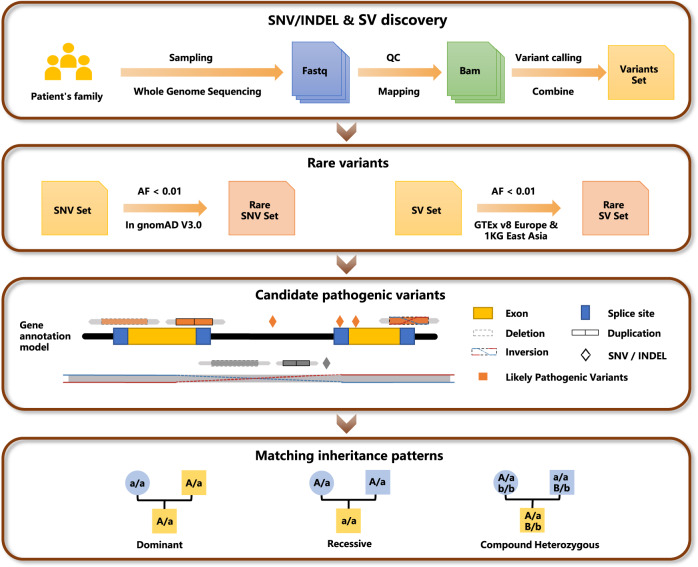
Flowchart for identifying pathogenic variants among an inherited retinal degeneration (IRD) cohort. After WGS sequencing and quality control, we performed SV and SNV/indel calling on a total of 271 patients together with their family members. Rare variants were identified by filtering allele frequencies with background populations. Variants were annotated against gene models considering retina-specific transcript expression. Candidate pathogenic variants were further examined for concordance with inheritance patterns among family members.

**Fig. 2:**
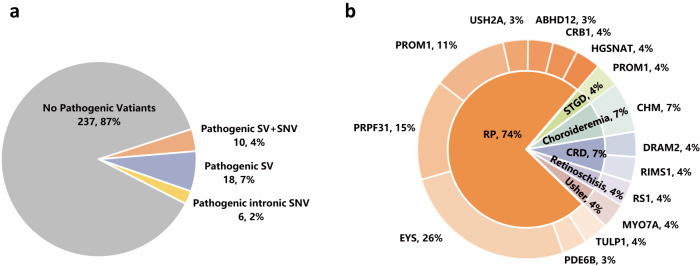
Summary of pathogenic variants identified in an IRD cohort. **a** WGS analysis in an IRD cohort achieved an overall diagnostic yield of 13%, among which 7% patients were caused by SVs only, 4% patients by a combination of SNVs and SVs while another 2% by intronic SNVs affecting splicing. **b** Distribution of 22 SVs and 5 intronic variants among 14 IRD genes, corresponding to 6 IRD disease groups.

A total of 271 patients with clinically suspected IRDs were recruited for SV and intronic variant analyses. All patients underwent an initial test with panel-based sequencing, but the genetic diagnosis was inconclusive. We performed WGS sequencing on these patients and relevant family members, identified both SNVs/indels and SVs. The pathogenicity of these variants was assessed through a comprehensive evaluation, considering allele frequency (AF < 0.01 in background populations, as shown in Supplementary Fig. 1), functional annotation, and inheritance patterns (as depicted in Fig. 1). Overall, pathogenic SVs and intronic variants were detected in 34 patients, yielding a diagnostic rate of 13% (as illustrated in Fig. 2a, Supplementary Fig. 2, and detailed in Supplementary Table 1). Among them, 13 patients had previously been found to carry a single variant each in a recessive gene by panel sequencing, including 3 splicing, 6 missense, and 1 nonsense variants across 7 IRD genes. The newly detected SVs and intronic variants were found in compound heterozygosity with these SNVs. In the remaining 21 patients, the definitive pathogenicity was attributed exclusively to the newly identified SVs and intronic variants. This included homozygous recessive SVs in 4 patients, heterozygous dominant SVs in 9 patients, compound heterozygous SVs in 2 patients, homozygous recessive intronic variants in 1 patient, and heterozygous dominant intronic variants in 2 patients. In addition, two hemizygous deletions in chromosome X were found in 3 male IRD cases. Of these patients with a confirmed genetic diagnosis, only 2 cases were sporadic, while the remaining cases were familial.

### Identification of SVs and intronic variants

Twenty-two SVs, 3 deep-intronic variants, and 2 non-canonical splice-site variants spanning 14 IRD genes were identified in the complete cohort (Tables 1 and 2), and all of these detected variants were novel. Among the SVs, with the exception of one inversion, all others were classified as large deletions. Of these variants, a significant majority, 74% (20/27) of variants were found in RP causative genes (*EYS*, *PRPF31*, *PROM1*, *USH2A*, *ABHD12*, *CRB1*, *HGSNAT*, *PDE6B*, and *TULP1*). The top one of 9 RP genes, *EYS*, accounted for 26% (*n* = 7) of all identified variants. In contrast, the remaining IRD genes responsible for CRD, STGD-like disease, retinoschisis, choroideremia, and Usher syndrome collectively accounted for only 26% (7/27) of variants (Fig. 2b). We observed five SVs and two deep-intronic variants within the *EYS* gene across five distinct families and one sporadic case. A deletion (chr4:633534–637421) in the *PDE6B* gene was found in 2 unrelated families, FM13 and FM105. Furthermore, our analysis revealed a deletion (chr4:15992516–15997089) involving the *PROM1* gene, which interestingly presented in two distinct disease phenotypes. Specifically, three patients displaying STGD-like phenotypes within family FM112 were identified as heterozygotes carrying this SV. In contrast, patients in FM289 and FM297, both diagnosed with RP, were found to carry the homozygous and compound heterozygous deletions (chr4:15992516–15997089), respectively (as illustrated in Supplementary Fig. 2).

**Table 1:** Overview of pathogenic SVs identified in IRD patients.

NO.	Gene	Disease/ Inheritance	SV	Zyg	Genomic Positions (hg38)	Consequence	First Report Study
1	*ABHD12*	RP/ AR	inv	Het	chr20:25313454–25555587	Exons 1-6 del	This study
2	*CHM*	Choroideremia/XL	del	Hem	chrX:85877526–85879620	Exon 13 del	This study
3	*CHM*	Choroideremia/XL	del	Hem	chrX:85939182–86023133	Exons 3-8 del	This study
4	*CRB1*	RP/ AR	del	Het	chr1:197259028–197272940	Exon 1 del	This study
5	*DRAM2*	CRD/ AR	del	Het	chr1:111124590–111125146	Exon 5 del	This study
6	*EYS*	RP/ AR	del	Het	chr6:64915646–65010155	Exons 14-15 del	This study
7	*EYS*	RP/ AR	del	Het	chr6:64389138–64488837	Exons 27-28 del	This study
8	*EYS*	RP/ AR	del	Het	chr6:64620376–64627760	Exon 23 del	This study
9	*EYS*	RP/ AR	del	Het	chr6:64911543–64913167	Exon 16 del	This study
10	*EYS*	RP/ AR	del	Het	chr6:64956270–65164613del	Exons 13-14 del	This study
11	*PDE6B*	RP/ AR	del	Hom	chr4:633534–637421	Exons 2-3 del	This study
12	*PRPF31*	RP/ AD	del	Het	chr19:54064664–54133055	Complete gene del	This study
13	*PRPF31*	RP/ AD	del	Het	chr19:54099322–54133113	Complete gene del	This study
14	*PRPF31*	RP/ AD	del	Het	chr19:53996498–54132343	Complete gene del	This study
15	*PRPF31*	RP/ AD	del	Het	chr19:54118226–54122901	Exons 2-5 del	This study
16	*PROM1*	RP/ AR	del	Het	chr4:16012199–16038605	Exons 3-10 del	This study
17	*PROM1*	RP/ AR	del	Het	chr4:16031002–16041704	Exons 2-4 del	This study
18	*PROM1*	RP/ AR	del	Hom/Het	chr4:15992516–15997089	Exon 15 del	This study
19	*PROM1*	STGD/ AD	del	Het	chr4:15992516–15997089	Exon 15 del	This study
20	*RS1*	Retinoschisis/ XL	del	Hem	chrX-18644599–18650206	Exons 4-5 del	This study
21	*TULP1*	RP/ AR	del	Het	chr6:35502609–35506996	Exons 8-12 del	This study
22	*USH2A*	RP/ AR	del	Het	chr1:216072986–216073478	Exon 28 del	This study

*Zyg* zygosity, *inv* inversion, *del* deletion, *Hem* hemizygous, *Het* heterozygous, *Hom* homozygous, *chr* chromosome.

**Table 2:** Intronic variants found in the study.

NO.	Gene	Disease/ Inheritance	Zyg	Genomic Positions (hg38)	cDNA variant	First Report Study
1	*EYS*	RP/ AR	Het	chr6:63964152 T > C	c.7055+20231 A > G	This study
2	*EYS*	RP/ AR	Het	chr6:65226028 A > C	c.2023+69835 T > G	This study
3	*HGSNAT*	RP/ AR	Het	chr8:43146937 A > G	c.119-11 A > G	This study
4	*MYO7A*	Usher syndrome / AR	Hom	chr11:77214594 C > G	c.6559-13 C > G	This study
5	*RIMS1*	CRD/ AD	Het	chr6:72106499 T > A	c.471+6513 T > A	This study

*Zyg* zygosity, *Het* heterozygous, *Hom* homozygous, *chr* chromosome.

**Fig. 3:**
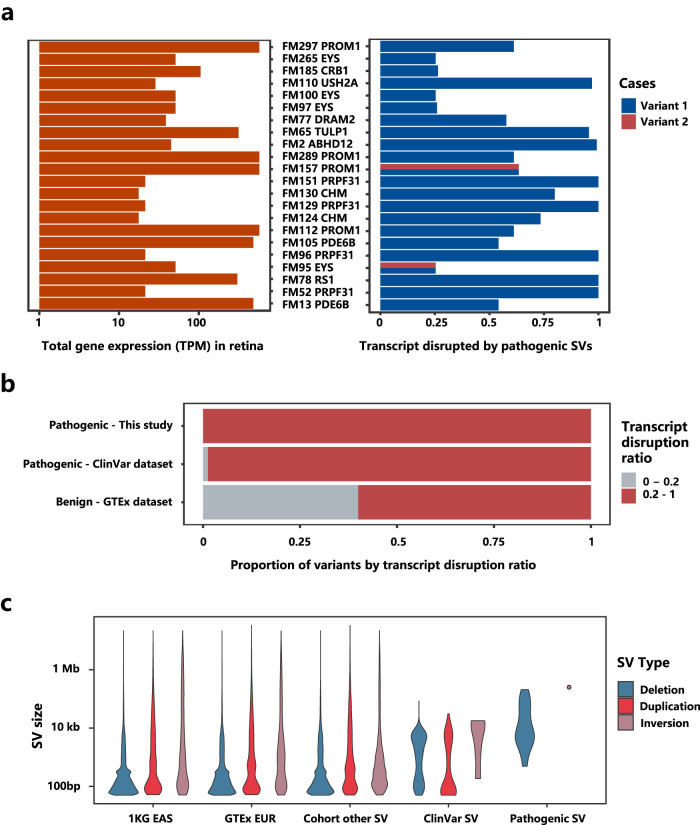
Pathogenic SV identification considering retina-specific transcript expression. **a** Expression levels (TPM) of SV-disrupted genes in retina (left panel) and transcript disruption ratio of pathogenic SVs (right panel). **b** Comparison of pathogenic and benign variants by transcript disruption ratio in retina. **c** Genomic features of pathogenic and sporadic SVs.

The retina has a very distinct gene expression profile compared to other tissues, making retina-specific transcripts highly informative in assessing variant pathogenicity among IRD cases. We quantified the “transcript disruption ratio” as the fraction of transcripts disrupted by these SVs to the total expression of all transcripts of a gene in the retina (Fig. 3a). We found that the vast majority of identified pathogenic SVs have a transcript disruption ratio of at least 20% in the retina, while SVs with a transcript disruption ratio below 20% were most likely benign (Fig. 3b). Among our cases, the lowest transcript disruption ratios of pathogenic SVs were observed in *EYS* and *CRB1*, standing at 25% and 26%, respectively (Fig. 3a, Supplementary Fig. 3). On the other hand, most benign variants would exhibit a retina-specific transcript disruption ratio below 20%. For example, we encountered one family harbored one inversion and one duplication in *RP1* (Supplementary Fig. 4), however these two variants only affected a transcript with 0.43 TPM, out of a total gene expression level of 361 TPM of *RP1* in the retina. These two variants are also carried by the patient’s father with no relevant phenotypes. Consequently, these variants were deemed nonpathogenic.

Further characterization of pathogenic SVs revealed that they were significantly larger in size compared to sporadically occurring SVs within the general population (Fig. 3c). Additionally, pathogenic SVs identified in our cohort exhibited greater length than those previously reported in ClinVar, indicating technological advancements enabled by WGS.

**Fig. 4:**
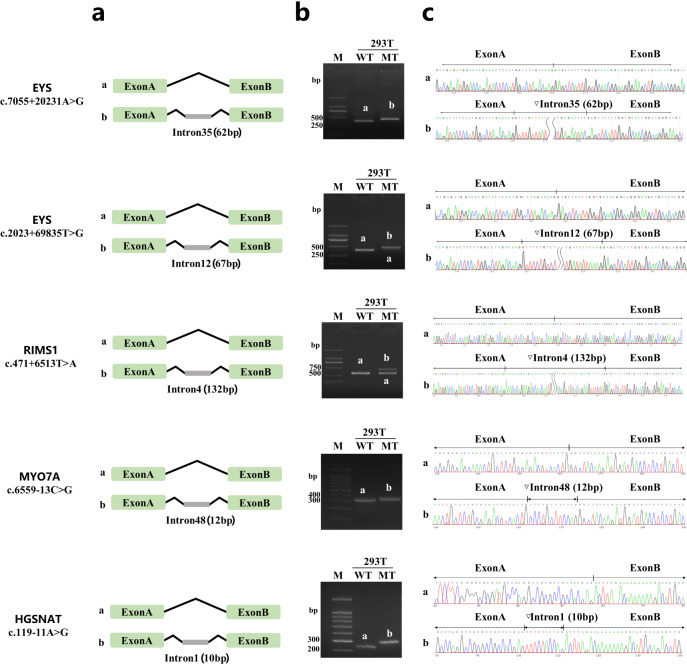
A minigene splicing assay reveals variant-induced aberrant splicing. **a** Diagram illustrating intron sequence retention caused by intronic variants. Gel electrophoresis (**b**) and Sanger sequencing (**c**) results of reverse transcription PCR (RT-PCR) products of all tested minigenes. WT wild-type, MT mutation-type, (**a**) splicing results of wild-type control; (**b**) splicing results of identified variants.

We verified the splicing-altering effect of 3 deep-intronic variants and 2 non-canonical splice-site variants through minigene assays (Fig. 4). All of these variants were found to cause the retention of intronic segments, resulting in the generation of premature termination codons (PTCs). As a consequence, these intronic variants induce loss-of-function effects on the genes they affect. Specifically, the deep-intronic variant *EYS* c.7055+20231A > G produced a 62 bp sequence within intron 35, the deep-intronic variant *EYS* c.2023+69835T > G generated a 67 bp sequence within intron 12, the deep-intronic variant *RIMS1* c.471+6513T > A resulted in a 132 bp sequence within intron 4, the non-canonical splice-site variant *MYO7A* c.6559-13C > G produced a 12 bp sequence within intron 48, and the non-canonical splice-site variant *HGSNAT* c.119-11A > G generated a 10 bp sequence within intron 1.

## Discussion

IRDs are a group of ophthalmic hereditary diseases with high genetic and clinical heterogeneity, and genetic testing has greatly assisted the clinical diagnosis of IRDs. A series of studies have demonstrated the contribution of SVs and intronic variants to the genetic diagnosis of IRDs^17,20,21^. In our current study, we identified 27 pathogenic SVs and intronic variants across 14 different IRD genes in 34 previously unresolved IRD cases through WGS. It is worth noting that all variants detected in these patients have not been previously reported, thus our study represents the first report of these specific variants, underscores the significance of SVs and intronic variants in IRDs.

In 271 IRD patients, the disease-associated SVs were identified in 11% of cases, in concordance with previous reports of the pathogenic proportion (5% to 15%) of SVs in the molecular diagnosis of IRDs^15^. It is worth noting that all cases included in this study had been previously screened by panel-based sequencing and showed negative results. Although panel sequencing also included CNV analysis, no large deletions or duplications suspected to be pathogenic were found in these cases. This underscores the potential of WGS as an optimal sequencing method for detecting SVs, offering a higher rate of genetic diagnosis. Notably, we observed that 4% of cases exhibited a compound heterozygous pathogenicity pattern involving both SVs and SNVs in recessive genes, highlighting a previously underexplored form of pathogenicity arising from the combined effects of SVs and SNVs. Such pathogenic patterns are often overlooked as SNV/indel and SV screening are typically conducted separately. Furthermore, in this study, intronic variants contributed an additional 2% to the overall pathogenicity, further affirming the diagnostic significance of deleterious variants located in non-coding regions, particularly those affecting mRNA splicing. Notably, all five of the intronic variants we identified had not been previously reported, suggesting that the contribution of deep-intronic variants to the molecular diagnosis of IRDs may have been underestimated in previous studies.

In total, 22 disease-associated SVs, 3 deep-intronic variants, and 2 non-canonical splice-site variants of IRD genes were found. Except for one inversion of *ABHD12*, SVs in the remaining 10 IRD genes were deletions (including homozygous, heterozygous, and hemizygous deletions), ranging from single exon to complete gene deletions. All detected SVs were defined as novel pathogenic variants, further extending the mutational spectrum of IRD genes. In this study, we confirmed that approximately three-quarters of SVs were clustered in RP-related genes, of which the most frequently altered genes by SVs were *EYS* (*n* = 5) and *PRPF31* (*n* = 4). Previous studies have highlighted *PRPF31*, *EYS*, and *USH2A* as the most prevalent pathogenic genes harboring SVs in IRDs^10^. A homozygous deletion (chr4:633534–637421) in *PDE6B* was reported for the first time in this study, which was concurrently observed in three RP patients from two unrelated families (FM13 and FM105), corroborating the pathogenic significance of this deletion. Moreover, 5 novel intronic variants, including deep-intronic variants and non-canonical splice-site variants, were detected from 4 IRD genes in 4 families and one sporadic case. These intronic variants identified were validated to cause aberrant splicing by minigene assays.

None of the detected pathogenic intronic variants and SVs were observed or had extremely low AFs in the general population. The AFs of SVs varied widely among different population groups, with a very limited sharing of SVs between European and East Asian populations (Supplementary Fig. 1). We therefore used diverse background population groups to confirm true rare SVs, as a prerequisite for establishing pathogenicity. Using an inadequate population background can lead to misinterpretation of rare variants, especially when analyzing small, sporadic cohorts. We therefore recommend the SV reference set to include at least one population group matching the patient cohort. Notably, most current SV annotation methods do not consider tissue-specific transcript information. Consequently, benign SVs that do not affect specific transcripts in disease-relevant tissues may be frequently mis-labeled as pathogenic. As shown in our study, pathogenic and benign SVs exhibited rather different transcript disruption ratios. Implementing a threshold of 20% for transcript disruption ratio significantly reduced false positives in the identification of pathogenic SV identification, particularly in sporadic cases.

We also conducted genotype-phenotype correlations among IRD patients. The average age of the probands was 35 years (range 8–58 years old) and the average age of onset was 15.0 years (range 2–50 years old). Variants in the *USH2A* gene result in either RP (OMIM 613809) or Usher syndrome (OMIM 276901)^22^. In our study, the ocular phenotypes of patients harboring *USH2A* variants were consistent with clinical manifestations of RP, which were characterized by progressive night blindness and reduced visual field. The fundus showed waxy optic disc, retinal osteocytes-like pigmentation, retinal vascular stenosis, accompanied by retinal atrophy and thinning. However, these patients did not display symptoms of diseases beyond ocular involvement and were ultimately diagnosed with RP rather than Usher syndrome. By contrast, patients carrying *MYO7A* variants exhibited both RP and hearing impairment, aligning with the diagnosis of Usher syndrome. Notably, the proband with *ABHD12* variants in FM2 had syndromic features, presenting with both characteristics of RP and deafness. We further checked the clinical phenotype of the remaining patients carrying variants in RP causative genes, and their symptoms and fundus manifestations were consistent with the clinical diagnosis of RP. The representative RP photographs from the proband in FM110 were shown in Supplementary Fig. 5. We detected variants in genes associated with CRD (*DRAM2* and *RIMS1*) in two families, FM77 and FM134. The probands presented with decreased visual acuity and abnormal color vision, with electroretinography (ERG) indicating more severe impairment of cone photoreceptor function than rod photoreceptor. Additionally, fundus examinations revealed macular atrophy. For example, the color fundus photograph of proband from FM134 displayed waxy optic disc discoloration, macular atrophy, and retinal vessel narrowing. Fundus autofluorescence (FAF) showed hypo-autofluorescence in the macular area surrounded by a hyperfluorescent ring. Spectral domain optical coherence tomography (SD-OCT) examinations revealed thinning of the macular fovea thickness, particularly in the neuroepithelial layer (Supplementary Fig. 6). According to the genetic test results of the probands, combined with the clinical phenotype and medical history, it was consistent with the diagnosis of CRD.

The clinical manifestations of IRDs are diverse, and variants in the same causative gene can lead to different clinical phenotypes^9^, posing challenges in the genetic diagnosis of IRD patients. Variants in *PROM1* are responsible for autosomal recessive or autosomal dominant IRDs, including STGD-like disease, RP, and CRD^23^. In our study, we observed *PROM1* deletions in 3 RP families and one family with STGD-like phenotypes. In families FM157, FM297, and FM289, compound heterozygous and homozygous deletions in *PROM1* were detected in probands, whereas their relatives with normal phenotype each carried a single heterozygous variant. Combining family history, AR mode of inheritance, clinical manifestation consistent with RP, as well as co-segregation analysis, the probands of the above three families were definitely diagnosed with RP. In FM112, the presence of a dominant heterozygous *PROM1* deletion in three patients resulted in the onset of STGD-like disease. The fundus of the patients displayed atrophy of the retinal pigment epithelium (RPE) in the macular area of both eyes. Fundus photographs of the proband in FM112 showed a “beaten bronze” atrophic area of the macula and yellow pisciform flecks in the posterior pole of the retina. Meanwhile, FAF examination clearly showed the range of macular lesions (hypo-autofluorescence), with pisciform hyperfluorescent dots observed around the macula. SD-OCT revealed the loss of outer retinal structures in the macular area, accompanied by RPE atrophy and thinning (Supplementary Fig. 7). Interestingly, the same deletion (chr4:15992516–15997089) in the *PROM1* gene was associated with two different clinical phenotypes (FM112, FM289, and FM297).

Moreover, due to overlapping phenotypes among various IRD conditions, accurate genetic diagnosis is crucial in refining clinical diagnoses for IRD patients^9^. For instance, patients in FM124 and FM130 were initially diagnosed with RP but were ultimately found to possess a hemizygous deletion in the *CHM* gene, the causative gene for choroideremia^24^. Advanced-stage choroideremia closely resembles end-stage RP, exhibiting similar chorioretinal atrophy and clinical symptoms, including night blindness and visual field constriction, with preserved central acuity. Genetic testing is imperative for a precise clinical diagnosis. Consequently, based on SV analysis of pathogenic genes, we conclusively diagnosed these two families with choroideremia.

In conclusion, our study highlights the potential of WGS to significantly enhance the diagnostic yield of IRDs and expand the mutational spectrum of known IRD-associated genes. The investigation of SVs and intronic variants holds substantial promise for the diagnosis and management of IRDs, facilitating personalized interventions for patients with these conditions.

## Methods

### Subjects and ethics declaration

A total of 271 IRD patients and their available family members (*n* = 646) were enrolled at Fudan University Eye Ear Nose and Throat Hospital from 2019 to 2020. All cases included in this study underwent a comprehensive ophthalmic examination and were given the diagnosis of IRDs by a professional ophthalmologist. Our research was approved by the Medical Ethics Committee of Fudan University Eye Ear Nose and Throat Hospital and in accordance with World Medical Association Code of Ethics on medical research involving human subjects (Declaration of Helsinki). Informed consent was signed by all subjects or parents on behalf of minors. Our study is performed in strict accordance with the ‘Guidance of the Ministry of Science and Technology (MOST) for the Review and Approval of Human Genetic Resources’.

### Read quality control and variants calling

All WGS Fastq files underwent quality control assessment using Fastqc (https://github.com/s-andrews/FastQC). Reads were aligned to the human reference genome build GRCh38 by BWA-MEM (http://bio-bwa.sourceforge.net). We retained bam files which satisfy: (1) mean sequence coverage >15; (2) percent of chimeric reads <0.05; (3) normal median and standard deviation of insert size as computed with Picard (http://broadinstitute.github.io/picard/); (4) contamination rate <0.05 estimated with VerifyBamID2 (http://griffan.github.io/VerifyBamID/).

SNVs and indels were called across WGS samples using GATK (https://gatk.broadinstitute.org/hc/en-us) HaplotypeCaller v4.2 and combined by GATK CombineGVCFs v4.2. Variant calling was restricted to autosomes and chromosome X. Variant QC was performed using GATK VQSR and Hail v0.2 (https://hail.is). Error-prone variant sites by any of the following criteria were filtered: (1) in Low Complexity Regions; (2) Inbreeding Coefficient < −0.3; (3) Hardy-Weinberg test <10–6; (4) failed VQSR at a sensitivity level below 99.8% for SNVs and indels. Low confidence genotypes were filtered: (1) read depth DP < 10 or DP > 400; (2) low genotype quality GQ < 25; (3) heterozygous calls of allelic imbalance HET AB < 0.25 or >0.75; and (4) homozygous calls of allelic imbalance HOM REF AB > 0.1 and HOM ALT AB < 0.9.

SVs were called by Manta^25^ for each sample. VCFs were combined by SURVIVOR^26^, where SVs of greater than 80% overlap were consolidated. We excluded from our SV calling regions of HLA, with decoy or alternate contigs and regions of much higher than the expected copy number^27^ (https://github.com/hall-lab/speedseq/blob/master/annotations/exclude.cnvnator_100bp.GRCh38.20170403.bed).

### Rare SNV/indel and SV identification

As SVs are abundant within human populations, effectively filtering for rare SVs is essential for identifying causal mutations within patient families. However, there is currently a lack of a homogenous SV reference panel for all population groups, whereas transferring SV callings from different pipelines presents inherent challenges. We therefore re-processed two large reference population sets of GTEx 831 European cohort^28^ and 1KG 196 East Asia cohort (https://www.internationalgenome.org/) using the same pipeline as our patient cohort to generate a consistent allele frequency (AF) estimate for SVs.

We defined SVs as rare when their AFs were below 1% in both populations. To filter for rare SNVs and indels, we used gnomAD3.0^29^ AF and gnomAD3.0 East Asian AF as our reference set. We required AF to be lower than 0.01 in both the overall population and the East Asian set.

### Variant annotation

We considered variants to be potentially pathogenic if occurring in coding or splice site regions of candidate genes (Gencode v38). To be deemed functionally damaging, a variant had to disrupt a highly expressed transcript in tissues associated with the relevant disease, which, in most cases, was retinal tissue. For SNVs and indels, we leveraged Ensembl VEP version 108 (https://grch37.ensembl.org/info/docs/tools/vep/index.html) to generate variant consequences. Additionally, we employed various tools and resources, including CADD v1.6^30^, SpliceAI^31^, EVE^32^, LOFTEE^29^ and CLINVAR annotations (https://www.ncbi.nlm.nih.gov/clinvar/) to prioritize SNVs and indels with potentially deleterious effects.

We defined variants in these categories as likely pathogenic. SV: (1) Deletion/Duplication: any overlap with an exon of a candidate gene; (2) Inversion: any overlap with an exon but not spanning a whole gene;

SNV/indel: (1) Stop gain: leading to the creation of a premature stop codon; (2) Stop loss: causing the loss of a stop codon; (3) Frameshift: reading frame disruption by indels; (4) Splice site variant: altering canonical splice donor or acceptor with SpliceAI score > 0.5 or CADD score > 20; (5) Missense: changing the coding amino acid and annotated as damaging by EVE or with CADD score > 20; (6) Deep-intronic variants: intronic variants with a distance ≥100 bp from the nearest exon and inferred to introduce cryptic splice sites (SpliceAI score > 0.5); (7) non-canonical splice-site variants: intronic variants with distance <100 bp from the nearest exon and inferred to introduce cryptic splice sites (SpliceAI score > 0.5).

### Candidate pathogenic genes and mutations identification

We established a candidate pathogenic gene list consisting of 792 genes associated with common hereditary ophthalmopathy. This list was compiled from sources such as OMIM (https://www.omim.org/) and published literature (Supplementary Table 2). The screening of candidate variants and genes was based on variant annotations and their known inheritance patterns in association with the relevant phenotypes. For dominant inheritance, we considered variants that were carried by the patients but not by their unaffected relatives. For recessive inheritance/compound heterozygous inheritance, we required that two pathogenic variants be carried by patients in the same gene but not by their unaffected relatives. We considered both inherited and de novo mutations, without requiring the parents of a patient to carry a same variant.

We further utilized retina-specific transcripts to differentiate benign SV events from pathogenic ones. We surveyed patterns of pathogenic and benign SVs by evaluating how they affect retina-specific transcripts. Pathogenic SVs were sourced from ClinVar’s collection of IRD pathogenic SVs (https://ftp.ncbi.nlm.nih.gov/pub/clinvar/vcf_GRCh38/clinvar_20230121.vcf.gz) and causal SVs in our IRD cohort. Benign SVs were identified from GTEx 831 individuals, meeting the following criteria: (i) overlapped with exons of candidate pathogenic genes, (ii) present in at least two samples and (iii) manually verified using the Integrated Genomics Viewer (IGV).

To evaluate the impact of SVs on retina-specific transcripts, we utilized RNA-seq data from 120 normal retinal samples^33^, which were part of GTEx external datasets. The quantification of retina-specific transcript expression was performed against the same gene model used in Gencode v38. For each SV, we defined a transcript disruption ratio as the summation of disrupted transcripts (in terms of expression level TPM), out of all transcripts of a gene. We noticed that SVs with a transcript disruption ratio below 20% were almost always benign. We therefore applied an empirical threshold of 20% to exclude benign SVs from our candidate pathogenic variants.

### Minigene molecular cloning, transfection, and reverse transcription PCR (RT-PCR)

To evaluate the potential pathogenic impact of variants on mRNA splicing, an in vitro approach based on minigene assays was designed. Introns harboring splice variants and control introns were amplified by PCR from genomic DNA using PrimerSTAR MAX DNA polymerase and oligonucleotide primer pairs (Supplementary Table 3). The wild type (WT) and mutant type (MT) minigenes were cloned into the pcMINI vector, which included a universal exon A-intron A-MCS-intron B-exon B construct. These vectors were subsequently transfected into 293 T cells by lipo3000 and harvested after 48 h; total RNA was extracted using Trizol (RNAiso PLUS) and then reverse transcribed to synthesize cDNA. RT-PCR products were separated by electrophoresis on a 2% agarose gel and subjected to Sanger sequencing for further analysis. All gels derived from the same experiment and they were processed in parallel.

### Reporting summary

Further information on research design is available in the Nature Research Reporting Summary linked to this article.

### Supplementary information

Supplementary Data

REPORTING SUMMARY

## Data Availability

The data used to support the findings of this study are available in the Supplementary Tables and deposited in the National Omics Data Encyclopedia (accession OEP004860). GTEx (v8) RNA-seq and WGS data are available from dbGaP (dbGaP: phs000424.v8.p2). GTEx (v8) summary statistics are obtained from the GTEx Portal available at https://gtexportal.org/home/datasets. 1000 Genomes data are available at http://www.internationalgenome.org.
