# Supplementary material for: Whole genome sequencing enables new genetic diagnosis for inherited retinal diseases by identifying pathogenic variants
Source: NPJ Genom Med. 2024 Jan 20;9:6. doi: 10.1038/s41525-024-00391-2 (PMC10799956; doi:10.1038/s41525-024-00391-2)
Supplement: Supplementary file 1 — Supplementary Data [file 41525_2024_391_MOESM1_ESM.pdf]

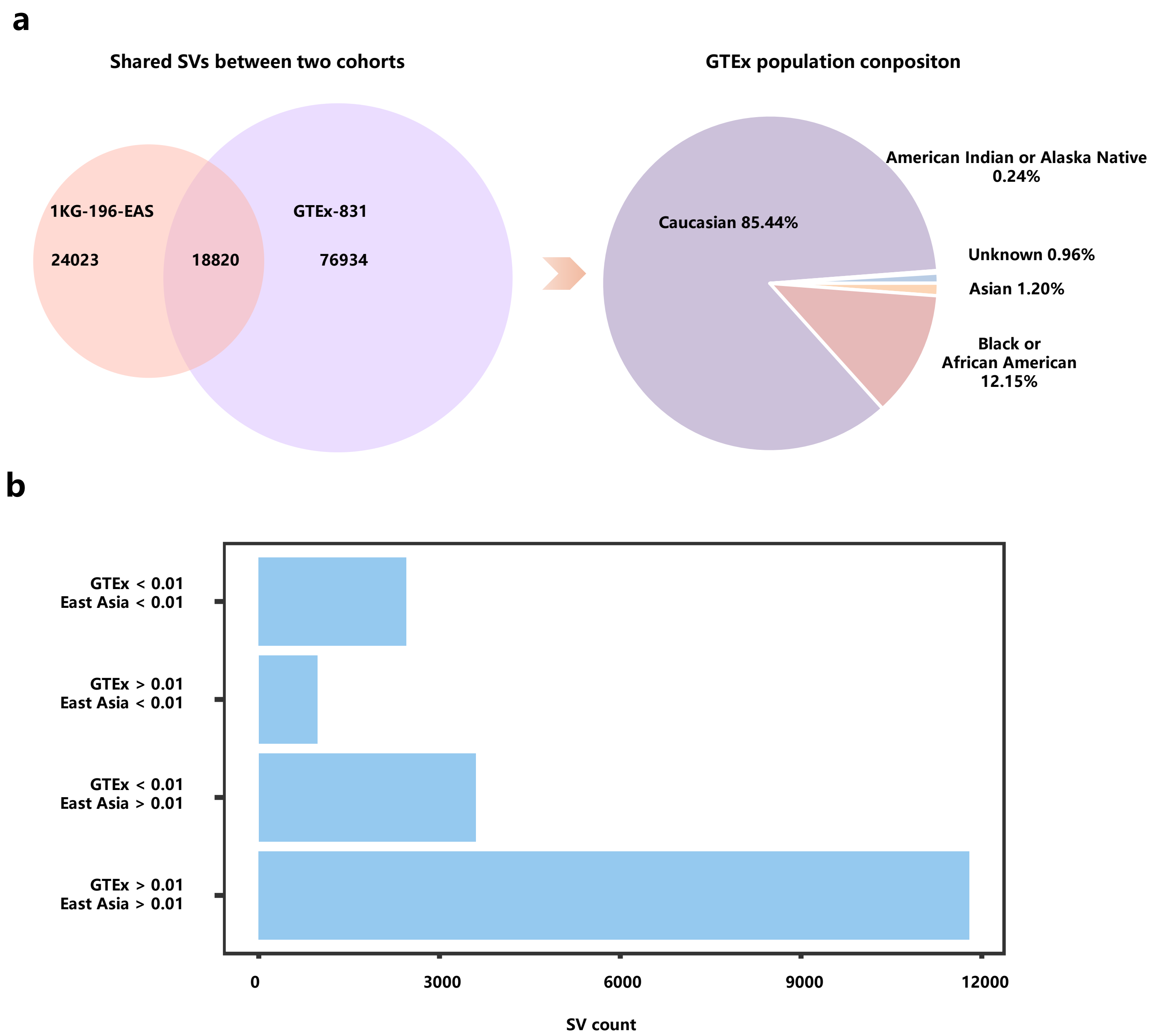

Supplementary Figure 1. Pathogenic SV identification considering population allele frequency heterogeneity. (a) Shared SVs between GTEx cohort (mainly of European ancestry) and 1KG East Asia cohort. The left pie chart indicates that the two cohorts share only a few SVs. The right pie chart illustrates the population composition of the GTEx cohort, with the largest population being Caucasian, but also having a small representation of individuals of Asian descent. (b) Distribution of SV allele frequency (AF) between two background populations. Y-axis represents SV allele frequency in different background populations, categorized as follows: a. SV AF < 0.01 in both cohorts; b. SV AF < 0.01 in 1KG East Asia cohort but > 0.01 in GTEx cohort; c. SV AF < 0.01 in GTEx cohort but > 0.01 in 1KG East Asia cohort; d. SV AF > 0.01 in both cohorts.

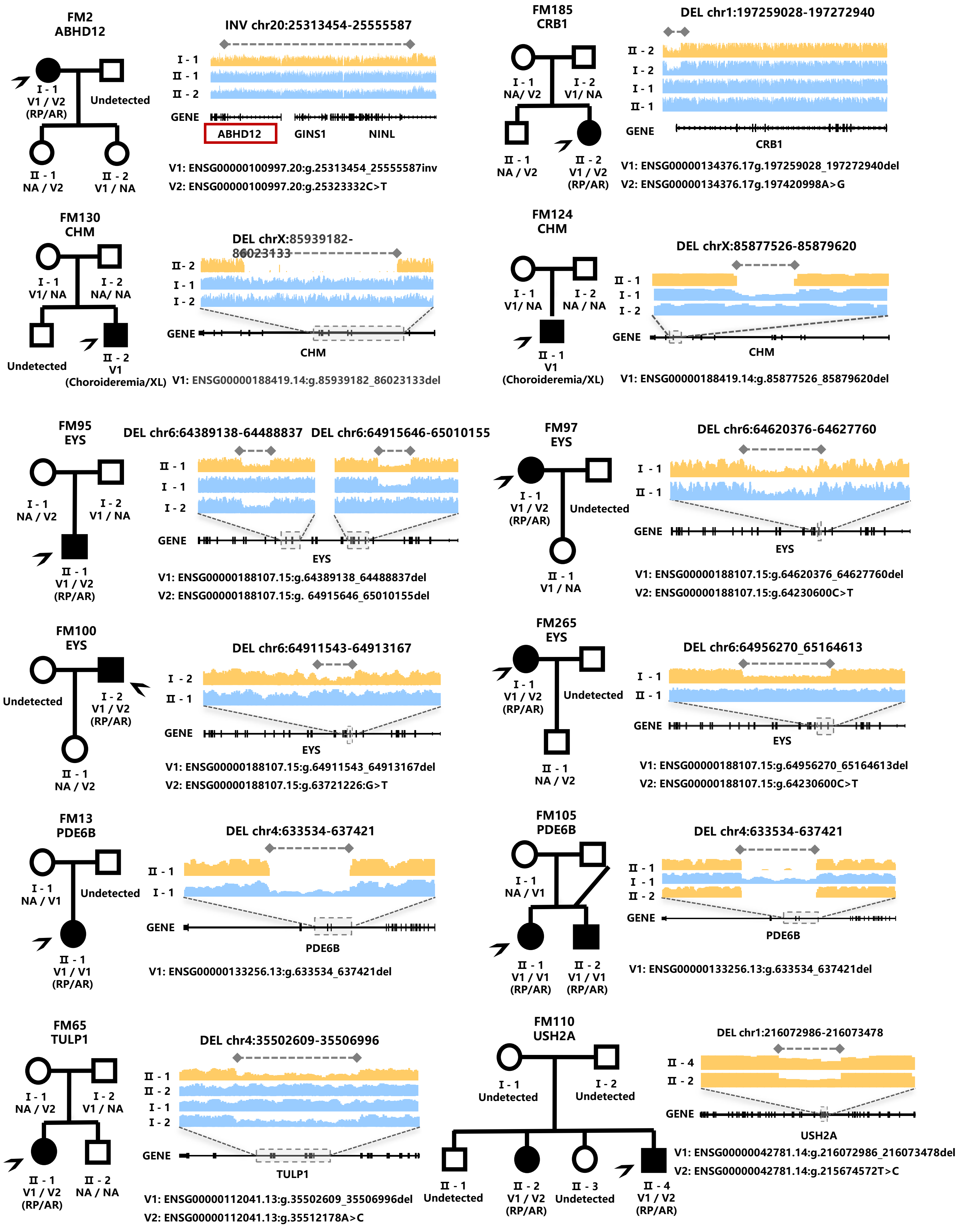

Supplementary Figure 2. The presentation of pathogenic variants in IRD cases with a definitive molecular diagnosis. Circles and squares represent females and males, respectively. Black filled shape symbols indicate patients, and blank shape symbols indicate family members with normal phenotypes. Double horizontal lines represent consanguineous marriages. Deceased family members are marked with slash lines. Black arrows refer to the probands in the pedigrees. "NA" stands for the wild-type allele and "V" for the mutant allele. All detected SVs are presented with visualized graphs.

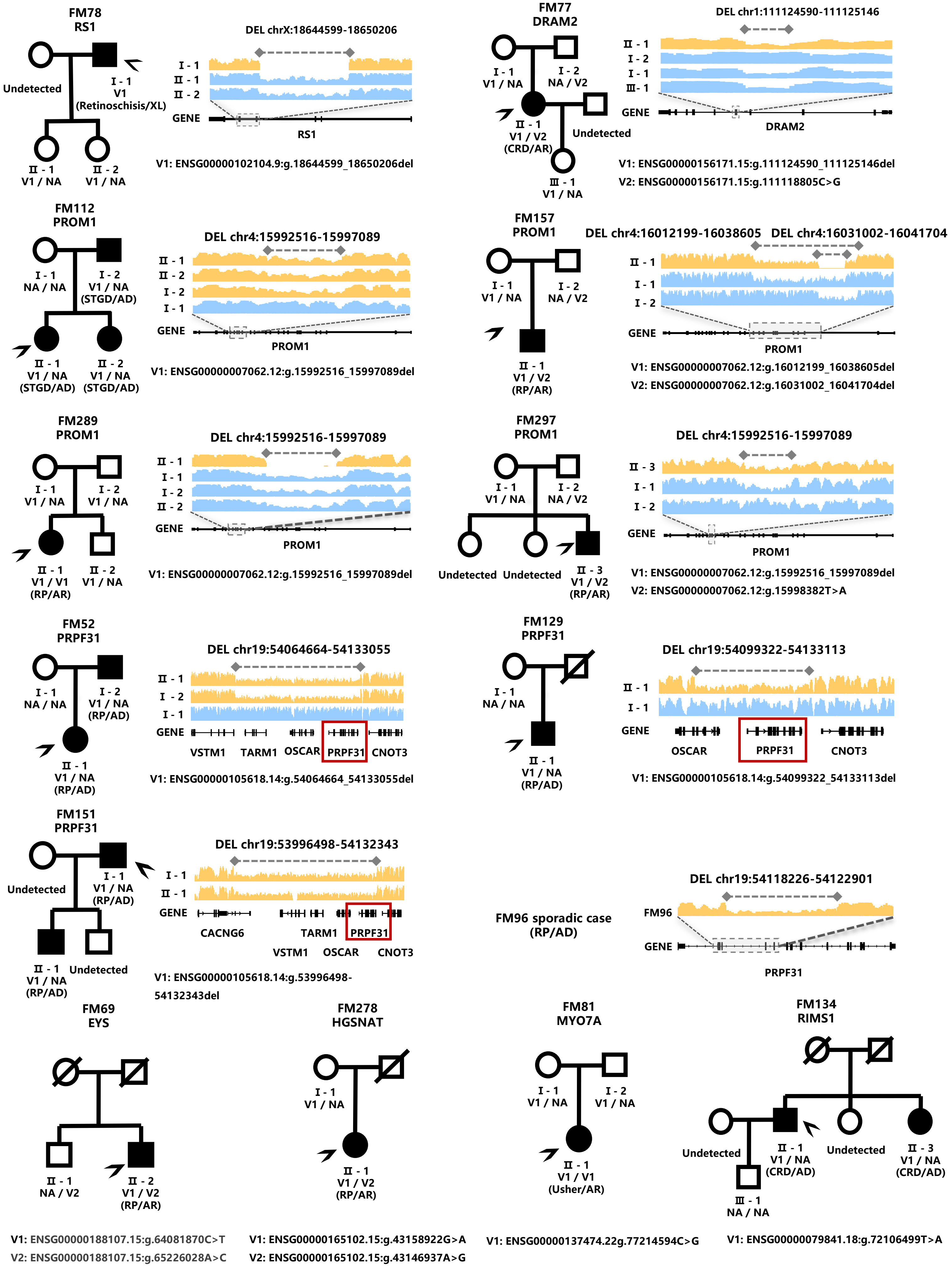

Supplementary Figure 2. The presentation of pathogenic variants in IRD cases with a definitive molecular diagnosis (continued). Circles and squares represent females and males, respectively. Black filled shape symbols indicate patients, and blank shape symbols indicate family members with normal phenotypes. Double horizontal lines represent consanguineous marriages. Deceased family members are marked with slash lines. Black arrows refer to the probands in the pedigrees. "NA" stands for the wild-type allele and "V" for the mutant allele. All detected SVs are presented with visualized graphs.

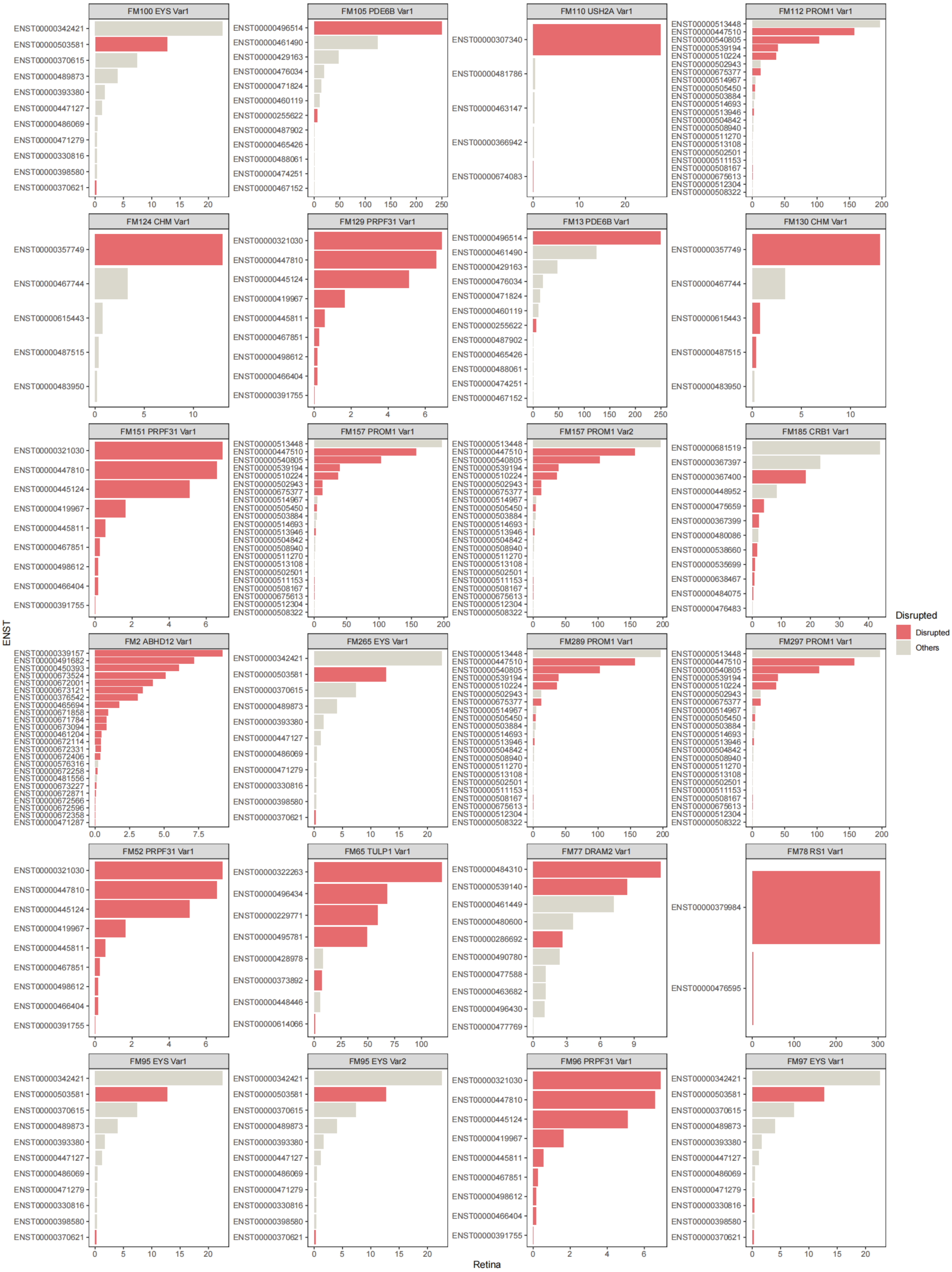

Supplementary Figure 3. Expression levels of disrupted transcripts in retina. The red bars indicate transcripts disrupted by pathogenic SVs.

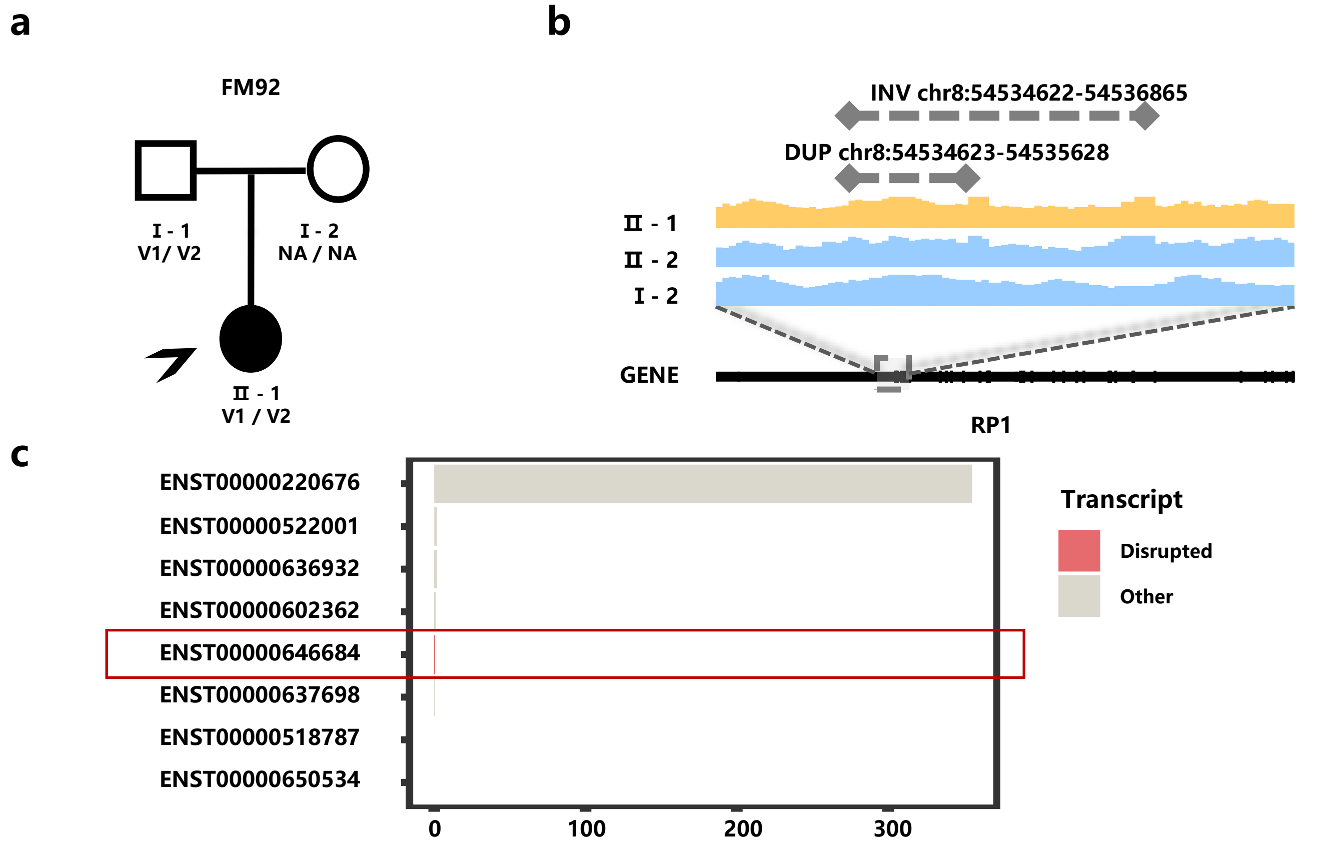

Supplementary Figure 4. Non-pathogenic variant case. (a) FM92 family. (b) SV track plot. (c) The SV disrupted transcript (red bar) of RP1 gene was lowly expressed in retina.

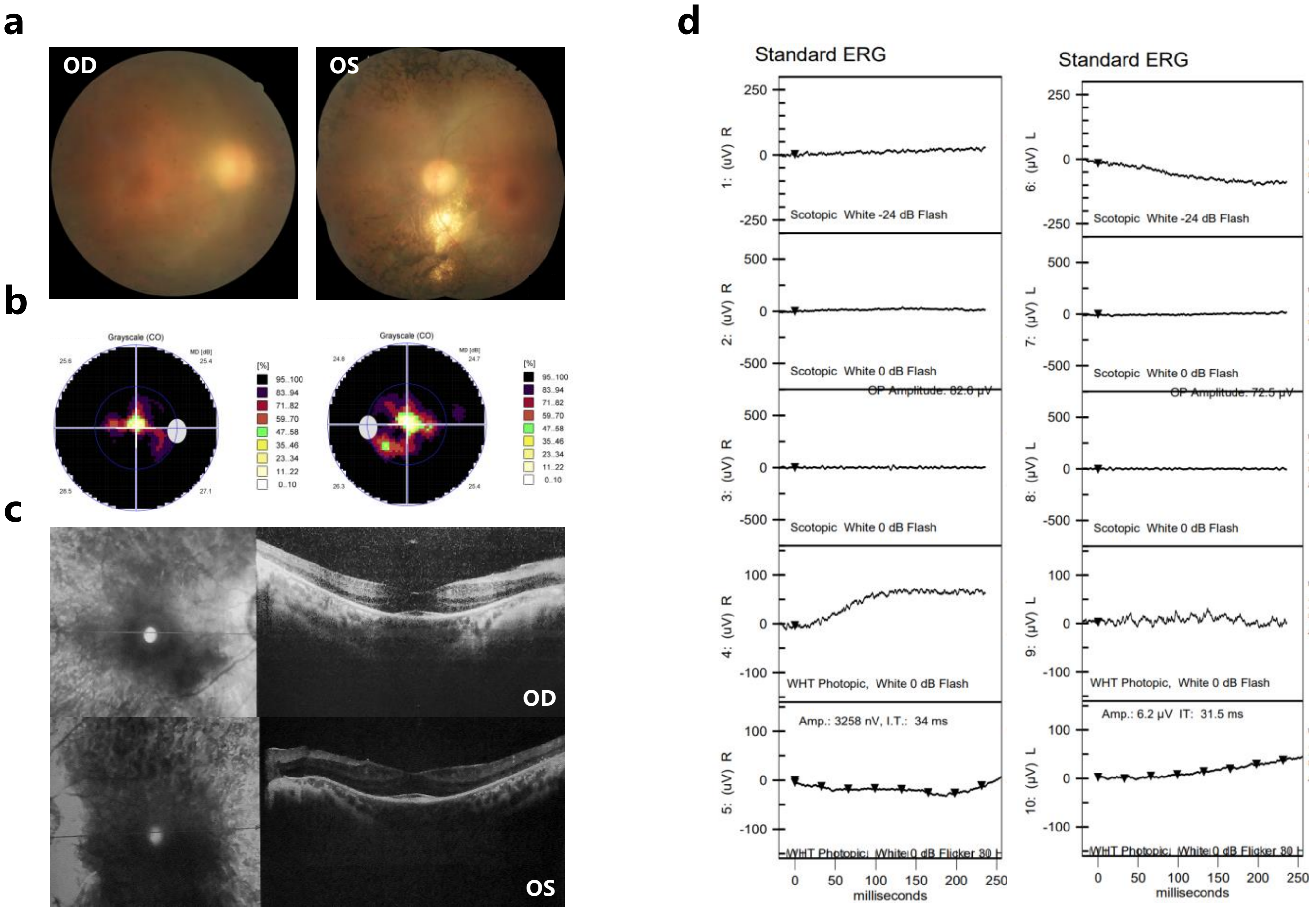

Supplementary Figure 5. Ocular features from proband in FM110. (a) Fundus photograph showing "osteocyte" like pigment deposits in the retina, arterial stenosis, and waxy optic disc atrophy. (b) Visual field showing small central field of both eyes. (c) Spectral domain optical coherence tomography (SD-OCT) showing atrophy and thinning of the retinal neuroepithelial layer. (d) The waveforms of electroretinography (ERG) disappeared in both eyes.

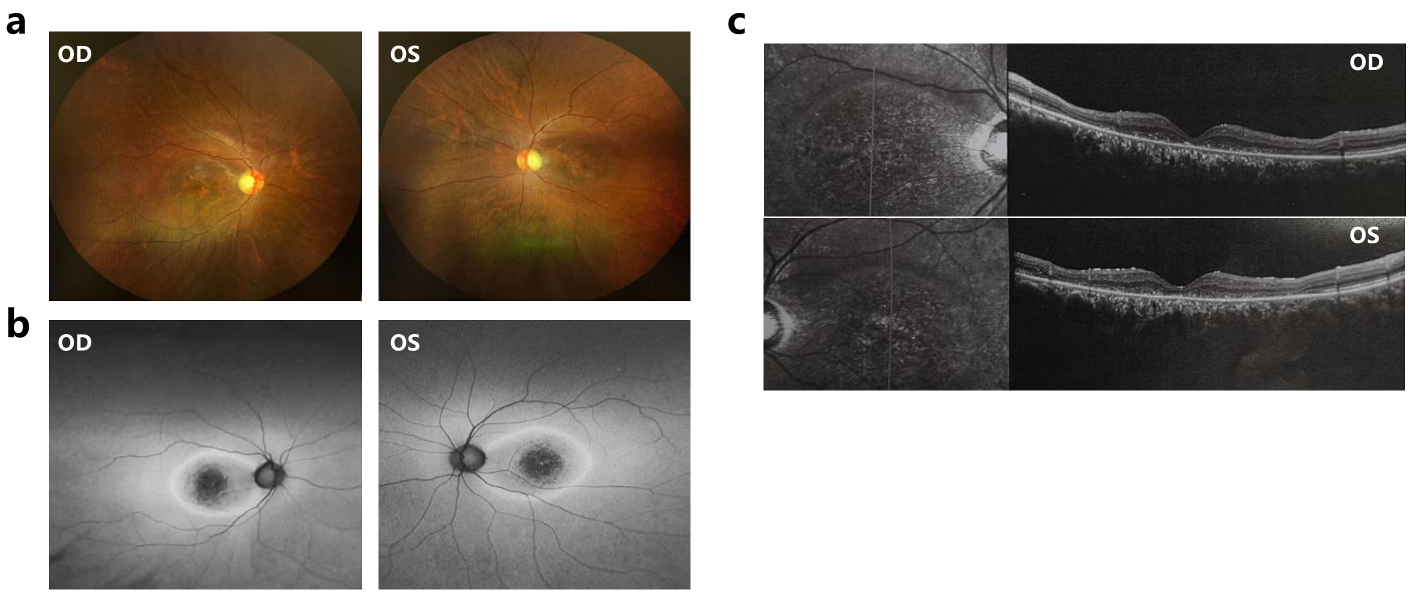

Supplementary Figure 6. Ocular features from proband in FM134. (a) Color fundus photograph showing waxy optic disc, retinal vessel narrowing, and macular atrophy in both eyes. (b) Fundus autofluorescence (FAF) exhibiting hypo-autofluorescence in the macular area surrounded by a hyperfluorescent ring. (c) SD-OCT showing thinning of the macular fovea thickness.

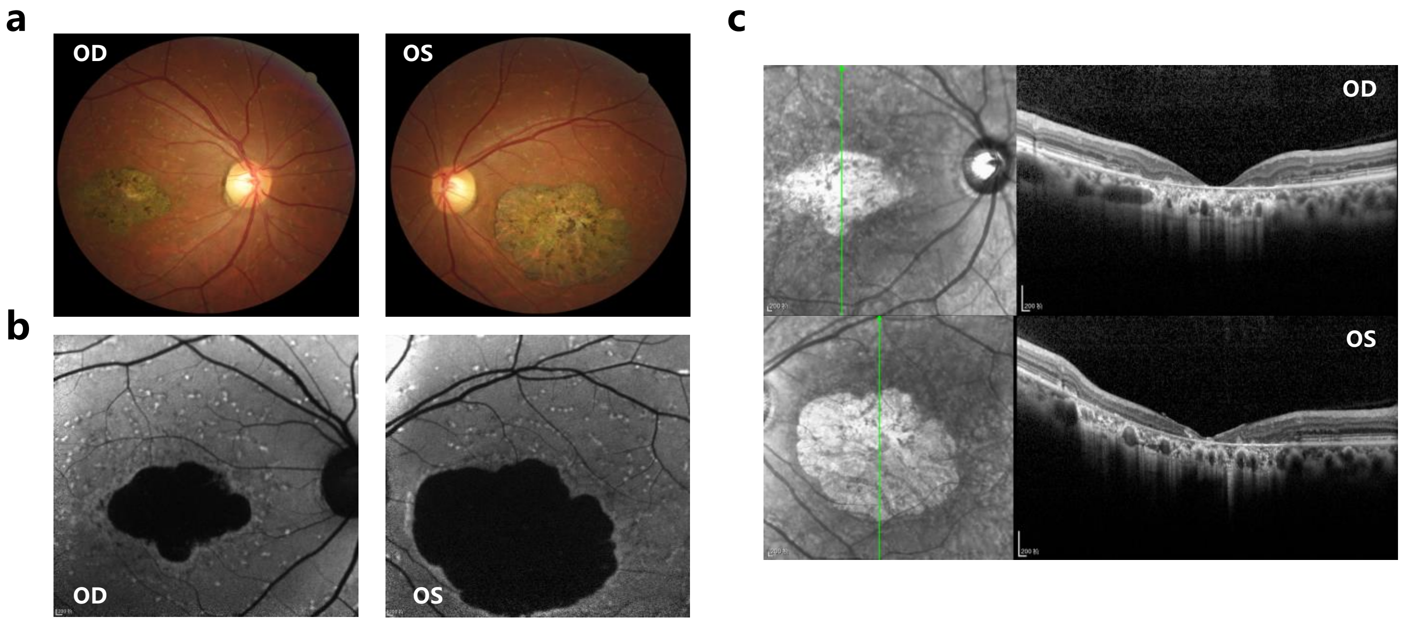

Supplementary Figure 7. Ocular features from proband in FM112. (a) Fundus photograph exhibiting bull's eye maculopathy caused by RPE atrophy, with yellow spots around the macular area. (b) FAF showing hypo-autofluorescence with a clear boundary in the macular area and hyperfluorescent dots corresponding with the surrounding yellow flecks. (c) SD-OCT showing thinning of retinal thickness in the fovea.

Supplementary Table 1. The disease-associated variants identified in probands with IRDs

ID	Disease	Inh	Gene	Chr	Variant ID	HGVSg	Zyg	Consequence	HGVSc	HGVSp	ACMG	Clinvar	LOVD	Co-segregation analysis
FM2	RP	AR	ABHD12	20	Var1	ENSG00000100997.20:g.25313454_25555587inv	Het	Exon truncating	-	-	PVS1+PS4+PM1+PM2+PM6+PP1+PP4	None	None	No
					Var2	ENSG00000100997.20:g.25323332C>T	Het	Missense	ENST00000339157.10:c.415G>A	ENSP00000341408.5:p.Gly139Arg	PS4+PM1+PM2+PM3+PP1+PP2+PP3+PP4	None	None	Yes
FM124	Choroideremia	XL	CHM	X	Var1	ENSG00000188419.14:g.85877526_85879620del	Hem	Exon truncating	-	-	PVS1+PS4+PM1+PM2+PP1+PP4	None	None	Yes
FM130	Choroideremia	XL	CHM	X	Var1	ENSG00000188419.14:g.85939182_86023133del	Hem	Exon truncating	-	-	PVS1+PS4+PM1+PM2+PP1+PP4	None	None	Yes
FM185	RP	AR	CRB1	1	Var1	ENSG00000134376.17g.197259028_197272940del	Het	Exon truncating	-	-	PVS1+PS4+PM1+PM2+PP1+PP4	None	None	Yes
					Var2	ENSG00000134376.17g.197420998A>G	Het	Splice acceptor site	ENST00000367400.8:c.1172-2A>G	-	PVS1+PS4+PM2+PP1+PP3+PP4+PP5	Likely_pathogenic	None	Yes
FM77	CRD	AR	DRAM2	1	Var1	ENSG00000156171.15:g.111124590_111125146del	Het	Exon truncating	-	-	PVS1+PS4+PM1+PM2+PP1+PP4	None	None	Yes
					Var2	ENSG00000156171.15:g.111118805C>G	Het	Missense+Splice Site	ENST00000484310.6:c.693G>C	ENSP00000503400.1:p.Gln231His	PVS1+PS4+PM1+PM2+PP1+PP2+PP3+PP4	None	None	Yes
FM15	RP	AR	EYS	6	Var1	ENSG00000188107.15:g.63789144C>G	Het	Missense	ENST00000503581.6:c.7492G>C	ENSP00000424243.1:p.Ala2498Pro	PS4+PM1+PM2+PM6+PP3+PP4+PP5	Pathogenic	Pathogenic	No, sporadic case
					Var2	ENSG00000188107.15:g.63964152T>C	Het	Deep intronic variant	ENST00000503581.6:c.7055+20231A>G	-	PVS1+PS3+PS4+PM2+PM3+PM6+PP3+PP4	None	None	
FM69	RP	AR	EYS	6	Var1	ENSG00000188107.15:g.64081870C>T	Het	Missense	ENST00000503581.6:c.6557G>A	ENSP00000424243.1:p.Gly2186Glu	PS4+PM1+PM2+PM6+PP1+PP3+PP4+PP5	Pathogenic	Pathogenic	No
					Var2	ENSG00000188107.15:g.65226028A>C	Het	Deep intronic variant	ENST00000503581.6:c.2023+69835T>G	-	PVS1+PS3+PS4+PM2+PM3+PM6+PP1+PP3+PP4	None	None	Yes
FM95	RP	AR	EYS	6	Var1	ENSG00000188107.15:g.64389138_64488837del	Het	Exon truncating	-	-	PVS1+PS4+PM1+PM2+PP1+PP4	None	None	Yes
					Var2	ENSG00000188107.15:g.64915646_65010155del	Het	Exon truncating	-	-	PVS1+PS4+PM1+PM2+PP1+PP4	None	None	Yes
FM97	RP	AR	EYS	6	Var1	ENSG00000188107.15:g.64620376_64627760del	Het	Exon truncating	-	-	PVS1+PS4+PM1+PM2+PM6+PP1+PP4	None	None	Yes
					Var2	ENSG00000188107.15:g.64230600C>T	Het	Missense	ENST00000503581.6:c.6416G>A	ENSP00000424243.1:p.Cys2139Tyr	PS4+PM1+PM2+PM6+PP1+PP3+PP4+PP5	Conflicting_interpretations_of_pathogenicity	Pathogenic	No
FM100	RP	AR	EYS	6	Var1	ENSG00000188107.15:g.64911543_64913167del	Het	Exon truncating	-	-	PVS1+PS4+PM1+PM2+PM6+PP1+PP4	None	None	No
					Var2	ENSG00000188107.15:g.63721226G>T	Het	Nonsense	ENST00000503581.6:c.8805C>A	ENSP00000424243.1:p.Ter2935%3D	PVS1+PS4+PM2+PM6+PP1+PP3+PP4+PP5	Pathogenic/Likely_pathogenic	Pathogenic	Yes
FM265	RP	AR	EYS	6	Var1	ENSG00000188107.15:g.64956270_65164613del	Het	Exon truncating	-	-	PVS1+PS4+PM1+PM2+PM6+PP1+PP4	None	None	No
					Var2	ENSG00000188107.15:g.64230600C>T	Het	Missense	ENST00000503581.6:c.6416G>A	ENSP00000424243.1:p.Cys2139Tyr	PS4+PM1+PM2+PM6+PP1+PP3+PP4+PP5	None	Pathogenic	Yes

Supplementary Table 1. The disease-associated variants identified in probands with IRDs (continued)

ID	Disease	Inh	Gene	Chr	Variant ID	HGVSg	Zyg	Consequence	HGVSc	HGVSp	ACMG	Clinvar	LOVD	Co-segregation analysis
FM278	RP	AR	HGSNAT	8	Var1	ENSG00000165102.15:g.43146937A>G	Het	Non-canonical splice-site variants	ENST00000379644.9:c.119-11A>G	-	PVS1+PS3+PS4+PM2+PM3+PM6+PP1+PP3+PP4	None	None	No
					Var2	ENSG00000165102.15:g.43158922G>A	Het	Splice acceptor site	ENST00000379644.9:c.372-1G>A	-	PVS1+PS4+PM2+PM6+PP1+PP3+PP4	None	None	Yes
FM81	Usher syndrome	AR	MYO7A	11	Var1	ENSG00000137474.22g.77214594C>G	Hom	Non-canonical splice-site variants	ENST00000409709.9:c.6559-13C>G	-	PVS1+PS3+PS4+PM2+PP1+PP3+PP4	Uncertain_significance	None	Yes
FM13; FM105	RP	AR	PDE6B	4	Var1	ENSG00000133256.13:g.633534_637421del	Hom	Exon truncating	-	-	PVS1+PS4+PM1+PM2+PM6+PP1+PP4	None	None	Yes
FM112	STGD	AD	PROM1	4	Var1	ENSG00000007062.12:g.15992516_15997089del	Het	Exon truncating	-	-	PVS1+PS4+PM1+PM2+PP1+PP4	None	None	Yes
FM157	RP	AR	PROM1	4	Var1	ENSG00000007062.12:g.16012199_16038605del	Het	Exon truncating	-	-	PVS1+PS4+PM1+PM2+PP1+PP4	None	None	Yes
					Var2	ENSG00000007062.12:g.16031002_16041704del	Het	Exon truncating	-	-	PVS1+PS4+PM1+PM2+PP1+PP4	None	None	Yes
FM289	RP	AR	PROM1	4	Var1	ENSG00000007062.12:g.15992516_15997089del	Hom	Exon truncating	-	-	PVS1+PS4+PM2+PP1+PP4	None	None	Yes
FM297	RP	AR	PROM1	4	Var1	ENSG00000007062.12:g.15992516_15997089del	Het	Exon truncating	-	-	PVS1+PS4+PM1+PM2+PP1+PP4	None	None	Yes
					Var1	ENSG00000007062.12:g.15998382T>A	Het	Splice donor site	ENST00000447510.7:c.1682+3A>T	-	PVS1+PS4+PM2+PM6+PP1+PP3+PP4	None	None	Yes
FM52	RP	AD	PRPF31	19	Var1	ENSG00000105618.14:g.54064664_54133055del	Het	Whole gene deletion	-	-	PVS1+PS4+PM1+PM2+PP1+PP4	None	None	Yes
FM96	RP	AD	PRPF31	19	Var1	ENSG00000105618.14:g.54118226-54122901del	Het	Exon truncating	-	-	PVS1+PS4+PM1+PM2+PM6+PP4	None	None	No, sporadic case
FM129	RP	AD	PRPF31	19	Var1	ENSG00000105618.14:g.54099322_54133113del	Het	Whole gene deletion	-	-	PVS1+PS4+PM1+PM2+PM6+PP1+PP4	None	None	No
FM151	RP	AD	PRPF31	19	Var1	ENSG00000105618.14:g.53996498-54132343del	Het	Whole gene deletion	-	-	PVS1+PS4+PM1+PM2+PM6+PP1+PP4	None	None	Yes
FM78	Retinoschisis	XL	RS1	X	Var1	ENSG00000102104.9:g.18644599_18650206del	Hem	Exon truncating	-	-	PVS1+PS4+PM1+PM2+PM6+PP1+PP4	None	None	Yes
FM134	CRD	AD	RIMS1	6	Var1	ENSG00000079841.18:g.72106499T>A	Het	Deep intronic variant	ENST00000521978.6:c.471+6513T>A	-	PVS1+PS3+PS4+PM2+PM6+PP1+PP3+PP4	None	None	Yes
FM65	RP	AR	TULP1	6	Var1	ENSG00000112041.13:g.35502609_35506996del	Het	Exon truncating	-	-	PVS1+PS4+PM1+PM2+PP1+PP4	None	None	Yes
					Var2	ENSG00000112041.13:g.35512178A>C	Het	Splice donor site	ENST00000229771.11:c.190+2T>G	-	PVS1+PS4+PM2+PM6+PP1+PP3+PP4	None	None	Yes
FM110	RP	AR	USH2A	1	Var1	ENSG00000042781.14:g.216072986_216073478del	Het	Exon truncating	-	-	PVS1+PS4+PM1+PM2+PM6+PP1+PP4	None	None	Yes
					Var2	ENSG00000042781.14:g.215674572T>C	Het	Missense	ENST00000307340.8:c.13339A>G	ENSP00000305941.3:p.Met4447Val	PS4+PM1+PM2+PM6+PP1+PP3+PP4+PP5	Conflicting_interpretations_of_pathogenicity	Pathogenic	Yes

Supplementary Table 2. List of candidate causative genes

All genes involved in common inherited eye diseases
SRY, ABCA4, ABCB6, ABCC2, ABCC6, ABHD12, ACBD5, ACO2, ACTA1, ACTB, ACTG1, ADAM9, ADAMTS10, ADAMTS17, ADAMTS18, ADAMTSL4, ADAR, ADGRA3, ADGRV1, ADIPOR1, AGBL5, AGK, AHI1, AIPL1, ALMS1, ANAPC1, ANO5, AP3B1, APTX, ARL13B, ARL2BP, ARL3, ARL6, ARMS2, ASRGL1, ATF6, ATP13A2, ATP2C1, ATXN10, ATXN7, B3GALNT2, B3GLCT, B4GAT1, B9D1, BAP1, BBIP1, BBS1, BBS10, BBS12, BBS2, BBS4, BBS5, BBS7, BBS9, BCOR, BEST1, BFSP1, BFSP2, BLOC1S3, BLOC1S6, BMP4, BRAF, C10orf11, C10orf2, C12orf65, C1QTNF5, C2, C21orf2, C2orf71, C3, C5orf42, C8orf37, C9, CA4, CABP4, CACNA1F, CACNA2D4, CAPN3, CAPN5, CAV3, CBS, CC2D2A, CDH23, CDH3, CDHR1, CEP164, CEP250, CEP290, CEP41, CERKL, CFB, CFH, CFI, CFL2, CHD7, CHM, CHMP4B, CHN1, CHST6, CIB2, CISD2, CLN3, CLN5, CLN6, CLN8, CLRN1, CLUAP1, CNBP, CNGA1, CNGA3, CNGB1, CNGB3, CNNM4, COL11A1, COL11A2, COL18A1, COL2A1, COL4A1, COL6A1, COL6A2, COL6A3, COL9A1, COL9A2, CRB1, CRX, CRYAA, CRYAB, CRYBA1, CRYBA2, CRYBA4, CRYBB1, CRYBB2, CRYBB3, CRYGB, CRYGC, CRYGD, CRYGS, CSPP1, CST3, CTC1, CTDP1, CTNNA1, CTSD, CTSF, CX3CR1, CYP1B1, CYP4V2, DCN, DHDDS, DHX38, DMD, DMPK, DNA2, DNAJC5, DRAM2, DRD5, DTHD1, DTNBP1, DUX4, DYSF, EDN3, EDNRB, EFEMP1, ELOVL4, EMC1, EMD, EPHA2, ERCC1, ERCC2, ERCC6, ERCC8, EXOSC2, EYS, FAM126A, FAM161A, FBLN5, FBN1, FGF10, FGFR1, FGFR2, FGFR3, FHL1, FKRP, FKTN, FLVCR1, FOXC1, FOXE3, FOXL2, FRAS1, FREM1, FREM2, FRMD7, FSCN2, FTL, FYCO1, FZD4, GABRB1, GALC, GCNT2, DF3, GDF6, GFER, GJA1, GJA3, GJA8, GJB2, GMPPB, GNAT1, GNAT2, GNB3, GNPTG, GP1BA, GPR143, GPR179, GRIP1, GRK1, GRM6, GRN, GUCA1A, GUCA1B, GUCY2D, HARS, HCCS, HESX1, HFE, HGSNAT, HK1, HMCN1, HMGB3, HMX1, HPS1, HPS3, HPS4, HPS5, HPS6, HSF4, HTRA1, IDH3B, IDUA, IFT140, IFT172, IFT27, IGBP1, IKBKG, IMPDH1, IMPG1, IMPG2, INPP5E, INVS, IQCB1, ISPD, ITGA2B, ITGA7, ITGB3, ITM2B, JAG1, JAM3, KCNJ10, KCNJ13, KCNV2, KCTD7, KERA, KIAA1549, KIF11, KIF21A, KIF7, KIT, KIZ, KLHL7, KMT2D, KRAS, KRT12, KRT3, LAMA1, LAMA2, LAMB2, LCA5, MTND1, LIM2, LMNA, LOXL1, LRAT, LRIT3, LRP5, LTBP2, LYST, LZTFL1, MAB21L2, MAF, MAK, MAP2K1, MAPKAPK3, MAPT, MC1R, MCOLN1, MERTK, MFN2, MFRP, MFSD8, MIP, MIR204, MITF, MKKS, MKS1, MT-ATP6, MTM1, MT-TH, MT-TL1, MTTP, MT-TP, MTTS2, MVK, MYH7, MYO7A, MYOC, MYOT, NBAS, NDP, NEB, NEK2, NEUROD1, NHS, NMNAT1, NOD2, NPHP1, NPHP3, NPHP4, NR2E3, NR2F1, NRAS, NRL, NTF4, NYX, OAT, OCA2, OCRL, OFD1, OPA1, OPA3, OPN1LW, OPN1MW, OPN1SW, OPTN, OR2W3, OTX2, PABPN1, PANK2, PAX2, PAX3, PAX6, PCDH15, PCYT1A, PDE6A, PDE6B, PDE6C, PDE6G, PDE6H, PDZD7, PEX1, PEX2, PEX7, PGK1, PHOX2A, PHYH, PIGL, PIKFYVE, PITPNM3, PITX2, PITX3, PLA2G5, PLEC, PLG, PLK4, PNPLA6, POC1B, POLG, POLG2, POMGNT1, POMGNT2, POMK, POMT1, POMT2, PPT1, PRCD, PRDM13, PRDM5, PROM1, PRPF3, PRPF31, PRPF4, PRPF6, PRPF8, PRPH2, PRPS1, PRSS56, PTPN11, RAB18, RAB28, RAB3GAP1, RAB3GAP2, RAF1, RARB, RAX, RAX2, RB1, RBP3, RBP4, RCBTB1, RD3, RDH11, RDH12, RDH5, RGR, RGS9, RGS9BP, RHO, RIMS1, RLBP1, ROM1, RP1, RP1L1, RP2, RP9, RPE65, RPGR, RPGRIP1, RPGRIP1L, RRM2B, RS1, RTN4IP1, RYR1, SAG, SALL1, SDCCAG8, SEMA4A, SEPN1, SETX, SGCA, SGCB, SGCD, SGCG, SHH, SHOX, SIL1, SIX6, SLC16A12, SLC24A1, SLC24A5, SLC25A4, SLC25A46, SLC26A4, SLC45A2, SLC4A11, SLC4A4, SLC7A14, SMOC1, SNAI2, SNRNP200, SOS1, SOX10, SOX2, SPATA7, SPP2, STRA6, SYNE1, SYNE2, TACSTD2, TBC1D20, TCAP, TCOF1, TCTN1, TCTN2, TCTN3, TDRD7, TEAD1, TFAP2A, TGFBI, TIMM8A, TIMP3, TINF2, TLR4, TMEM126A, TMEM138, TMEM216, TMEM231, TMEM237, TMEM5, TMEM67, TNNT1, TOPORS, TP63, TPM2, TPM3, TPP1, TREX1, TRIM32, TRIM37, TRNT1, TRPM1, TSPAN12, TTC21B, TTC8, TTLL5, TTN, TTPA, TTR, TUB, TUBA8, TUBB3, TUBGCP4, TUBGCP6, TULP1, TYR, TYRP1, UBIAD1, UNC119, USH1C, USH1G, USH2A, VAX1, VCAN, VIM, VSX1, VSX2, WDPCP, WDR19, WDR36, WFS1, ZNF408, ZNF423, ZNF469, ZNF513, ABCA3, ABHD5, ACD, ACVRL1, AFG3L2, AGPS, AGRN, AGXT, ALDH18A1, ALDH1A3, , ALDH3A2, ANO10, ANTXR1, AP4M1, AP5Z1, APC, ARSB, ARSE, ASAH1, ASB10, ASPM, ATL1, ATM, ATP6V0A2, ATP7A, ATP7B, AUH, B4GALNT1, BLM, C19orf12, CCM2, CDK5RAP2, CENPJ, CEP135, CEP152, CHAT, CHMP1A, CHRNG, CLCN7, COASY, COL3A1, COL4A4, COL4A5, COLEC11, COLQ, COX10, COX15, COX7B, CREBBP, CTSA, CUBN, CYLD, CYP27A1, CYP2U1, CYP7B1, DAG1, DBH, DDHD2, DDX59, DHCR7, DHODH, DNAJC19, DNM2, DOK7, EBP, EDARADD, EEF2, EFEMP2, EP300, EPG5, ERCC3, ERLIN2, ESCO2, EXOSC3, FA2H, FAH, FLNA, FMR1, FUCA1, GALE, GALK1, GALNS, GALT, GBA, GBA2, GCM2, GDF2, GFAP, GLB1, GM2A, GNAS, GNPAT, GNS, GRHPR, GUSB, HDAC8, HEXA, HGD, HOGA1, HPD, HSPD1, HSPG2, HYAL1, IDS, IKBKAP, IRF6, ITPR1, KCNC3, KCND3, KCNH2, KCNJ2, KCNQ1, KDM6A, KIAA0196, KIF1A, KIF1BP, KIF5A, LAMA3, LAMB3, LAMC2, LARGE1, LCAT, LMX1B, LRPAP1, LTBP4, MAN2B1, MANBA, MARS2, MCPH1, MID1, MLPH, MMACHC, MRE11A, MSH2, MTPAP, NAGLU, NEU1, NF2, NFIX, NHP2, NIPA1, NIPBL, NOP10, NOTCH2, NPC1, NPC2, NSD1, OSTM1, PDK3, PEX10, PEX11B, PEX12, PEX13, PEX14, PEX16, PEX26, PEX5, PEX6, PHF6, PIK3R1, PKP1, PLA2G6, PLOD1, POLR1C, PORCN, PRIMPOL, PRKCG, PRX, PTCH1, PTCH2, PTH, PYCR1, RAB27A, RAD21, RARS2, RECQL4, REEP1, RNASEH1, ROBO3, RTN2, SALL4, SCN4A, SEPSECS, SF3B4, SLC33A1, SLITRK6, SMC1A, SMPD1, SNX10, SOD2, SPG11, SPG7, SPINK5, SPTBN2, STIL, STK11, STS, SURF1, SYT14, TACO1, TAT, TBX1, TCIRG1, TERT, TFAP2B, TGFBR1, TGM6, TLR1, TLR2, TNFRSF11A, TNFSF11, TNXB, TRPV4, TSC1, TSC2, TSEN2, TSEN34, TSEN54, TTBK2, TWIST1, UCHL1, UROD, VHL, VRK1, WHRN, WRAP53, WRN, WWOX, XPA, XPC, ZFYVE26, ZFYVE27, ZNF335, ZNF644, ACTA2, ATP1A3, CAV1, CHRDL1, CNTN1, COL8A2, FBN2, IARS2, LONP1, MPZ, MSMO1, MSTN, MYH11, MYLK, NAA10, P3H2, PRKG1, PXDN, SEMA3E, SLC2A10, SMAD3, TCF4, TGFB2, TGFB3, TGFBR2, ZEB1

Supplementary Table 3. Minigene primers sequence

Gene	HGVSg	HGVSc	Primer name	Sequence(5'-3')
EYS	ENSG00000188107.15:g.63964152T>C	c.7055+20231A>G	EYS-F1	ggtaGGTACCgttttgctctatttgccttg
			EYS-R1	tttcCTCGAGcacagactatgggttttcat
EYS	ENSG00000188107.15:g.65226028A>C	c.2023+69835T>G	EYS-F2	ggtaGGTACCcagtaattctatttttaact
			EYS-R2	TGCAGAATTCgcagcatattaaaaatggta
RIMS1	ENSG00000079841.18:g.72106499T>A	c.471+6513T>A	RIMS1-F	ggtaGGTACCcaactgtctagtaattcagc
			RIMS1-R	TAGTGGATCCaagccaggtgcttatcagag
HGSNAT	ENSG00000165102.15:g.43146937A>G	c.119-11A>G	HGSNAT-F	aatGCGGCCGCATGAGCGGGGCGGGCAGGGC
			HGSNAT-R	GAAATTAGGAAAAGTGCCGGGCGGCGGTAG
MYO7A	ENSG00000137474.22g.77214594C>G	c.6559-13C>G	MYO7A-F	aatGCGGCCGCCATCTGATGCCTTCTCATCT
			MYO7A-R	aatCTCGAGGGCATGGAACCAGCACCTCC
