# Supplementary material for: Whole genome sequencing enables new genetic diagnosis for inherited retinal diseases by identifying pathogenic variants
Source: NPJ Genom Med. 2024 Jan 20;9:6. doi: 10.1038/s41525-024-00391-2 (PMC10799956; doi:10.1038/s41525-024-00391-2)
Supplement: Supplementary file 2 — REPORTING SUMMARY [file 41525_2024_391_MOESM2_ESM.pdf]

nature portfolio

Corresponding author(s): Jihong Wu

Last updated by author(s): Nov 6, 2023

Reporting Summary

Nature Portfolio wishes to improve the reproducibility of the work that we publish. This form provides structure for consistency and transparency in reporting. For further information on Nature Portfolio policies, see our Editorial Policies and the Editorial Policy Checklist.

Statistics

For all statistical analyses, confirm that the following items are present in the figure legend, table legend, main text, or Methods section.

n/a	Confirmed	
☐	☒	The exact sample size (*n*) for each experimental group/condition, given as a discrete number and unit of measurement
☒	☐	A statement on whether measurements were taken from distinct samples or whether the same sample was measured repeatedly
☐	☒	The statistical test(s) used AND whether they are one- or two-sided<br>*Only common tests should be described solely by name; describe more complex techniques in the Methods section.*
☒	☐	A description of all covariates tested
☐	☒	A description of any assumptions or corrections, such as tests of normality and adjustment for multiple comparisons
☐	☒	A full description of the statistical parameters including central tendency (e.g. means) or other basic estimates (e.g. regression coefficient) AND variation (e.g. standard deviation) or associated estimates of uncertainty (e.g. confidence intervals)
☒	☐	For null hypothesis testing, the test statistic (e.g. *F*, *t*, *r*) with confidence intervals, effect sizes, degrees of freedom and *P* value noted<br>*Give P values as exact values whenever suitable.*
☒	☐	For Bayesian analysis, information on the choice of priors and Markov chain Monte Carlo settings
☒	☐	For hierarchical and complex designs, identification of the appropriate level for tests and full reporting of outcomes
☒	☐	Estimates of effect sizes (e.g. Cohen's *d*, Pearson's *r*), indicating how they were calculated

Our web collection on statistics for biologists contains articles on many of the points above.

Software and code

Policy information about availability of computer code

Data collection	No software was used.
Data analysis	No software was used.

For manuscripts utilizing custom algorithms or software that are central to the research but not yet described in published literature, software must be made available to editors and reviewers. We strongly encourage code deposition in a community repository (e.g. GitHub). See the Nature Portfolio guidelines for submitting code & software for further information.

Data

Policy information about availability of data

All manuscripts must include a data availability statement. This statement should provide the following information, where applicable:

- Accession codes, unique identifiers, or web links for publicly available datasets
- A description of any restrictions on data availability
- For clinical datasets or third party data, please ensure that the statement adheres to our policy

Whole-genome sequencing data from the current study are subject to conditions of Ministry of Science and Technology (China) policies on the management of human genetic resources under which the data was generated, and therefore the raw sequencing data is unavailable. These datasets can only become available upon a data sharing agreement approved by Ministry of Science and Technology (China). The additional variant requests or other data can be obtained from the corresponding author on reasonable request.

Research involving human participants, their data, or biological material

Policy information about studies with human participants or human data. See also policy information about sex, gender (identity/presentation), and sexual orientation and race, ethnicity and racism.

Reporting on sex and gender	They are not been considered in study design and our article does not cover this part.
Reporting on race, ethnicity, or other socially relevant groupings	Our article does not cover this part.
Population characteristics	Chinese population with inherited retinal diseases. The average age of the probands was 35 years (range 8–58 years old) and the average age of onset was 15.0 years (range 2–50 years old).
Recruitment	A total of 271 IRD patients and their available family members (n=646) were enrolled at Fudan University Eye Ear Nose and Throat Hospital from 2019 to 2020. All cases included in this study underwent a comprehensive ophthalmic examination and were given the diagnosis of IRDs by a professional ophthalmologist.<br><br>No potential self-selection bias or other biases are present.
Ethics oversight	Our research was approved by the Medical Ethics Committee of Fudan University Eye Ear Nose and Throat Hospital and in accordance with World Medical Association Code of Ethics on medical research involving human subjects (Declaration of Helsinki). Informed consent was signed by all subjects or parents on behalf of minors. Our study is performed in strict accordance with the 'Guidance of the Ministry of Science and Technology (MOST) for the Review and Approval of Human Genetic Resources'.

Note that full information on the approval of the study protocol must also be provided in the manuscript.

Field-specific reporting

Please select the one below that is the best fit for your research. If you are not sure, read the appropriate sections before making your selection.

☒ Life sciences ☐ Behavioural & social sciences ☐ Ecological, evolutionary & environmental sciences

For a reference copy of the document with all sections, see nature.com/documents/nr-reporting-summary-flat.pdf

Life sciences study design

All studies must disclose on these points even when the disclosure is negative.

Sample size	A total of 271 IRD patients and their available family members (n=646) were enrolled. All patients underwent an initial test with panel-based sequencing, but the genetic diagnosis was inconclusive. So, we performed WGS sequencing on these patients and relevant family members to identify both SNVs/indels and SVs.
Data exclusions	No data were excluded from the analyses.
Replication	The reproducibility of the minigene experimental findings has been verified. The results of minigene were confirmed by gel electrophoresis and Sanger sequencing simultaneously.
Randomization	The item is not relevant to our study.
Blinding	The item is not relevant to our study.

Reporting for specific materials, systems and methods

We require information from authors about some types of materials, experimental systems and methods used in many studies. Here, indicate whether each material, system or method listed is relevant to your study. If you are not sure if a list item applies to your research, read the appropriate section before selecting a response.

Materials & experimental systems

n/a	Involved in the study
☒	☐ Antibodies
☒	☐ Eukaryotic cell lines
☒	☐ Palaeontology and archaeology
☒	☐ Animals and other organisms
☒	☐ Clinical data
☒	☐ Dual use research of concern
☒	☐ Plants

Methods

n/a	Involved in the study
☒	☐ ChIP-seq
☒	☐ Flow cytometry
☒	☐ MRI-based neuroimaging

Plants

Seed stocks	The item is not relevant to our study.
Novel plant genotypes	The item is not relevant to our study.
Authentication	The item is not relevant to our study.
